# Prevalence of peripheral arterial disease in high-risk patients using ankle-brachial index in general practice: a cross-sectional study

**DOI:** 10.1111/j.1742-1241.2008.01953.x

**Published:** 2009-01

**Authors:** P Cacoub, J-P Cambou, S Kownator, J-P Belliard, J-P Beregi, A Branchereau, P Carpentier, P Léger, F Luizy, D Maïza, E Mihci, M-A Herrmann, P Priollet

**Affiliations:** 1 Pierre et Marie Curie (Paris 6) UniversityParis, France; 2 Department of Internal Medicine, Pitié-Salpêtrière HospitalParis, France; 3 Rangueil Hospital, University of MedicineToulouse, France; 4 CardiologistThionville, France; 5 Department of Vascular Surgery, Geoffroy Saint Hilaire ClinicParis, France; 6 Department of Cardiovascular Imaging, Cardiologic Hospital, University HospitalLille, France; 7 Department of Vascular Surgery, La Timone HospitalMarseille, France; 8 Clinic of Vascular Medicine, University HospitalGrenoble, France; 9 Department of Cardiology, Pasteur ClinicToulouse, France; 10 AngeiologistParis, France; 11 Department of Thoracic and Cardiovascular Surgery, Côte de Nacre HospitalCaen, France; 12 sanofi-aventis FranceParis, France; 13 Bristol-Myers Squibb, Rueil-MalmaisonFrance; 14 Department of Vascular Medicine, Saint Joseph HospitalParis, France

## Abstract

**Aims:**

The deleterious nature of peripheral arterial disease (PAD) is compounded by a status of underdiagnosed and undertreated disease. We evaluated the prevalence and predictive factors of PAD in high-risk patients using the ankle-brachial index (ABI).

**Methods:**

The ABI was measured by general practitioners in France (May 2005–February 2006) in 5679 adults aged 55 years or older and considered at high risk. The primary outcome was prevalence of PAD (ABI strictly below 0.90).

**Results:**

In all, 21.3% patients had signs or symptoms suggestive of PAD, 42.1% had previous history of atherothrombotic disease and 36.6% had two or more cardiovascular risk factors. Prevalence of PAD was 27.8% overall, ranging from 10.4% in patients with cardiovascular risk factors only to approximately 38% in each other subgroup. Prevalence differed depending on the localization of atherothrombotic events: it was 57.1–75.0% in patients with past history of symptomatic PAD; 24.6–31.1% in those who had experienced cerebrovascular and/or coronary events. Regarding the classical cardiovascular risk factors, PAD was more frequent when smoking and hypercholesterolemia history were reported. PAD prevalence was also higher in patients with history of abdominal aortic aneurysm, renal hypertension or atherothrombotic event. Intermittent claudication, lack of one pulse in the lower limbs, smoking, diabetes and renovascular hypertension were the main factors predictive of low ABI.

**Conclusions:**

Given the elevated prevalence of PAD in high-risk patients and easiness of diagnosis using ABI in primary care, undoubtedly better awareness would help preserve individual cardiovascular health and achieve public health goals.

**What’s known**

Peripheral arterial disease (PAD) is an atherothrombotic syndrome associated with elevated risk of cardiovascular and cerebrovascular events. Its deleterious nature is compounded by a status of underdiagnosed and undertreated disease. Ankle-brachial index (ABI) measurement easily provides reliable data for diagnosis of PAD. In practice, however, diagnosis is still usually based on the presence of symptoms.

**What’s new**

The ABI was measured in 5679 high-risk patients by general practitioners in France. Prevalence of PAD was high (28%) and ranged from 10% in patients with cardiovascular risk factors only to 38% in other (symptomatic or not) high-risk patients. Factors predictive of low ABI were identified. Better awareness of such elevated prevalence would help preserve individual health and achieve public health goals.

## Introduction

Clinical manifestations of lower extremity peripheral arterial disease (PAD) are associated with decrements in functional capacity and quality of life, cause limb amputation and increase risk of death (1,2). The systemic nature of the atherosclerotic process also contributes to the development of concomitant disease of the arteries to the heart and brain, hence to an associated increased risk of cardiovascular ischaemic events, such as myocardial infarction, ischaemic stroke and cardiovascular death. Even, vascular morbidity and mortality are similar in patients with symptomatic or asymptomatic PAD (3). Individuals with asymptomatic PAD should be identified so that therapeutic interventions known to diminish their increased risk of ischaemic events may be offered (1). Early diagnosis and treatment of PAD are increasingly recognised as a health burden worldwide (4).

The ankle-brachial index (ABI) is a measurement that provides objective data for the diagnosis of PAD. The overall accuracy of the ABI to establish PAD diagnosis has been well-established against contrast angiography and Doppler ultrasound (5–8) and is considered to have good reproducibility (9–12). It is usually obtained by measuring the resting systolic blood pressure (SBP) from the brachial, dorsalis pedis and posterior tibial arteries; dividing in each leg, the highest of the ankle pressures by the highest of the brachial pressures; then taking the lowest result (13,14). Besides, it is the recommended standard for PAD diagnosis; a low ABI is consistently associated with increased mortality and risk of cardiovascular disease (2). Early detection of PAD using ABI at least in patients considered at high risk should allow preventive intervention taking place earlier and improve cardiovascular outcome. However, although easily assessable, the ABI is largely underused in office practice and the deleterious nature of PAD is compounded by a status of underdiagnosed and undertreated disease (15).

Knowledge of the epidemiology of PAD arises from prevalence studies carried out in the USA or in various European countries, based on the presence of intermittent claudication for the oldest studies (16,17) or on the ABI measurement for the most recent evaluations (18–22). The only prevalence study in France was performed 10 years ago (23). We present the results of a recent, large study carried out in France to evaluate the prevalence and predictive factors of PAD using the ABI in general practice, in patients considered at high risk.

## Methods

General practitioners in France, randomly selected from a national database, were proposed to participate in this cross-sectional study. Those who agreed to participate received a specific training to perform ABI measurements under standardised condition. Training was carried out by experienced angiologists and cardiologists during study workshops.

The first five consecutive patients aged 55 years or older were recruited if they were considered at high risk for PAD, irrespective of the reason for seeing the doctor. Three clinical, mutually exclusive, subgroups at high risk were predefined (17,18,20,24–26) as follows. Group 1 – One or more signs or symptoms suggestive of PAD: first evidence of intermittent claudication; atypical pain in a leg muscle (calf, thigh or buttock) while walking; pulseless lower limb artery (dorsalis pedis, posterior tibial, popliteal or femoral); iliac or femoral bruits and/or lower limb ulcer or gangrene. Group 2 – History of one or more atherothrombotic events (secondary prevention): myocardial infarction (Q wave or non-Q wave); angina pectoris (stable or unstable); stroke or ischaemic attack; previously established symptomatic PAD (Leriche-Fontaine stage II or higher) and/or carotid, coronary or lower limb artery revascularization (bypass graft or angioplasty). Group 3 – Two or more cardiovascular risk factors only without history of atherothrombotic event (primary prevention): history of smoking, hypercholesterolemia, diabetes and/or hypertension.

Patients’ demographic characteristics, body weight and height, signs or symptoms suggestive of PAD, cardiovascular medical and surgical history were searched by physical examination, by questioning and from the medical record. Smoking history was established in patients currently or formerly smoking at least one cigarette per day. Hypercholesterolemia was defined as a fasting low-density lipoprotein (LDL) cholesterol concentration of 1.3 g/l (3.4 mmol/l) or more on two occasions within the past year. The most recent concentrations of total, LDL and high-density lipoprotein (HDL) cholesterol were also retrieved. Diabetes was defined, regardless of whether it was type 1 or type 2, as a fasting glycaemia of 1.26 g/l or more on two occasions and/or use of diabetes medication. Arterial hypertension was diagnosed as a SBP of 140 mmHg or more, a diastolic blood pressure of 90 mmHg or more on two occasions and/or use of treatment.

### Outcome measures

The primary outcome measure was the ABI. Measurements were performed under standardised condition by the general practitioners. A Doppler ultrasonic pen device (8 mHz, Mini Dopplex® D900/EZ8; Huntleigh Healthcare Inc., Eatontown, NJ) was used with a standard sphygmomanometer at each site. With each subject in supine position and after a 5-minute rest, SBP was recorded in the right upper extremity at the brachial artery and in both lower extremities at the posterior tibial and dorsalis pedis arteries. We choose deliberately the most sensitive method for calculating the ABI. General practitioners calculated the ABI by dividing the lowest of the four ankle systolic pressures by the brachial systolic pressure.

### Sample size and statistical analysis

The primary outcome was the prevalence of PAD as defined by an ABI strictly below 0.90, which is known to vary between 5 and 80% depending on the risk profile and the threshold ABI chosen (23). Assuming that the smallest subgroup of interest would represent approximately 7% of the cohort, we determined that a sample of 8571 (600/0.07) patients was required to observe a prevalence of 10–50% with a precision of ± 2.5–4.0%.

Standard descriptive statistics were provided for all variables; 95% confidence intervals (CIs) were calculated when appropriate. Proportions were calculated taking into account missing data in the denominator. Groups were compared using the χ^2^ test. Factors predictive of PAD were investigated by multivariate logistic regression with backward elimination (at the 0.20 level) in the population of patients without previously established symptomatic PAD, regardless of the univariate analyses results. The following variables were entered in the initial model: patients’ characteristics (gender, age, body mass index, symptoms suggestive of PAD); traditional cardiovascular risk factors (hypertension, hypercholesterolemia, diabetes, smoking status, brachial SBP at the visit and latest measurement of LDL cholesterol); history of atherothrombotic events [abdominal aortic aneurysm, renovascular hypertension (renal artery stenosis), angina pectoris or myocardial infarction or coronary artery angioplasty/bypass graft, stroke, carotid artery angioplasty/bypass graft]. The corresponding odds ratios and their 95% CI were calculated. Statistical significance was accepted at the two-sided 0.05 level. Data were analysed using sas® 8.02 (SAS Institute Inc., Cary, NC).

## Results

### Patients’ characteristics

Between 2 May 2005 and 15 February 2006, 1219 general practitioners well-distributed throughout the nation included 5679 consecutive patients aged 55 or above, who they considered were at high risk for PAD. Of these, 1209 (21.3%) patients belonged to group 1; 2393 (42.1%) to group 2; 2077 (36.6%) to group 3. Table 1 summarises patient characteristics in the overall cohort and in these predefined subgroups.

**Table 1:** Main patient characteristics in the cohort and in predefined clinical subgroups. Values are numbers (percentages) of patients unless otherwise specified

Characteristics	All patients (*n* = 5679)	1. Symptoms suggestive of PAD (*n* = 1209)	2. Manifestation of atherothrombotic disease (*n* = 2393)	3. Cardiovascular risk factors only (*n* = 2077)
Mean (SD) age, year	69.1 (8.7)	68.8 (8.8)	71.0 (8.9)	67.2 (8.0)
Males	3593 (63.3)	699 (57.8)	1755 (73.3)	1139 (54.8)
Mean (SD) body mass index, kg/m^2^	27.6 (4.6)	27.6 (4.8)	27.1 (4.3)	28.2 (4.7)
Leg pain while walking	1975 (34.8)	734 (60.7)	995 (41.6)	246 (11.8)
Intermittent claudication	864 (15.2)	305 (25.2)	559 (23.3)	0 (0)
Pulseless dorsalis pedis and/or posterior tibial artery	1596 (28.1)	770 (63.7)	826 (34.5)	0 (0)
**Cardiovascular history**				
Angina pectoris	1274 (22.4)	0 (0)	1274 (53.2)	0 (0)
Myocardial infarction	762 (13.4)	0 (0)	762 (31.8)	0 (0)
Ischaemic stroke	365 (6.4)	0 (0)	365 (15.3)	0 (0)
Previously established symptomatic PAD	554 (9.8)	0 (0)	554 (23.2)	0 (0)
Symptomatic heart failure	385 (6.8)	58 (4.8)	290 (12.1)	37 (1.8)
Lower limb artery bypass graft or angioplasty	312 (5.5)	0 (0)	312 (13.0)	0 (0)
Coronary artery bypass graft or angioplasty	876 (15.4)	0 (0)	876 (36.6)	0 (0)
Carotid bypass graft or angioplasty	186 (3.3)	0 (0)	186 (7.8)	0 (0)
Abdominal aortic aneurysm	133 (2.3)	14 (1.2)	101 (4.2)	18 (0.9)
Renovascular hypertension (renal artery stenosis)	89 (1.6)	10 (0.8)	67 (2.8)	12 (0.6)
Lower limb amputation	20 (0.4)	0 (0)	20 (0.8)	0 (0)
**Cardiovascular risk factors**				
Hypertension	4593 (80.9)	907 (75.0)	1824 (76.2)	1862 (89.6)
Hypercholesterolemia	4201 (74.0)	777 (64.3)	1762 (73.6)	1662 (80.0)
Diabetes	2056 (36.2)	437 (36.1)	729 (30.5)	890 (42.9)
Smoking	1292 (22.8)	334 (27.6)	470 (19.7)	488 (23.5)
None	205 (3.6)	59 (4.6)	146 (6.1)	0 (0)
Two or more	4711 (83.0)	898 (74.3)	1736 (72.5)	2077 (100)

PAD, peripheral arterial disease.

Patients considered at high risk were mainly men (63.3%) with hypertension (80.9%) and/or hypercholesterolemia (74.0%); 22.8% had a smoking history. In group 1, 60.7% of patients had leg pain while walking; it was intermittent claudication in less than half of the patients. A majority of patients in group 2 had history of coronary artery disease (angina pectoris 53.2% and/or myocardial infarction 31.8%). The cardiovascular risk factors most frequently reported in group 3 were hypertension (89.6%), hypercholesterolemia (80.0%) and to a lower extent diabetes (42.9%). Distribution of risk factors was roughly similar in all subgroups.

History of PAD (without lower limb revascularization and/or amputation) was recorded in 302 patients overall. Among them, 212 (70.2%) had intermittent claudication, 205 (73.2%) had at least one pulseless lower limb artery and 200 (68.7%) had a pulseless dorsalis pedis and/or posterior tibial artery.

### Prevalence of PAD in the cohort (univariate analyses)

**Figure 1:**
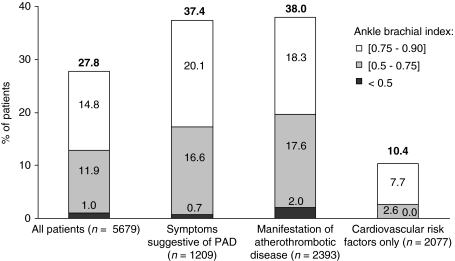
Prevalence of peripheral arterial disease (ankle-brachial index < 0.90) in the cohort (*n* = 5679) and in the predefined clinical subgroups

Prevalence of PAD was 27.8% (95% CI: 26.6–28.9) in the overall cohort (Figure 1). The ABI was below 0.50 in 1.0% of patients only. Mean duration of ABI measurement was 11.5 ± 6.06 min (median 10.0).

Univariate analyses (Table 2) showed increased prevalence of PAD in men, patients with older age or patients with history of coronary artery disease. PAD was approximately three times more frequent when at least one dorsalis pedis or tibial artery pulse was absent, or intermittent claudication was present, compared with when these symptoms were absent. To a lesser extent, prevalence of PAD was enhanced when brachial SBP was 160 mmHg or above at the visit, patients had a smoking history, history of hypercholesterolemia was absent, or recent HDL cholesterol levels were below 1 mmol/l (p < 0.001). PAD was also more frequent in patients with history of renal hypertension (52.8 vs. 27.4%, p < 0.0001) or abdominal aortic aneurysm (36.8 vs. 27.5%, p < 0.05). There was no significant impact of history of hypertension, history of diabetes or recent LDL cholesterol levels.

**Table 2:** Prevalence of peripheral arterial disease (ankle-brachial index < 0.9) according to patient characteristics, in the cohort and in predefined clinical subgroups. Univariate analysis

		All patients (*n* = 5679)		1. Symptoms suggestive of PAD (*n* = 1209)		2. Manifestation of atherothrombotic disease (*n* = 2393)		3. Cardiovascular risk factors only (*n* = 2077)	
Characteristic		Prevalence (%)	p-value	Prevalence (%)	p-value	Prevalence (%)	p-value	Prevalence (%)	p-value
**Demography**									
Gender	Male	30.2	< 0.0001	37.5	ns	40.4	< 0.0001	10.1	ns
	Female	23.5		37.3		31.3		10.7	
Age (year)	(55–64)	24.3	< 0.0001	37.7	ns	35.6	ns	9.4	ns
	(64–74)	27.1		36.2		38.9		10.3	
	≥ 74	31.6		38.3		38.6		12.1	
**Vascular history**									
Coronary artery disease	Yes	33.0	< 0.0001	na	na	33.0	< 0.0001	na	na
	No	25.5				50.4			
Ischaemic stroke	Yes	27.7	ns	na	na	27.7	< 0.0001	na	na
	No	27.8				39.8			
**Signs/Symptoms of PAD**									
Intermittent claudication	Yes	65.9	< 0.0001	52.1	< 0.0001	73.3	< 0.0001	na	na
	No	20.9		32.4		27.2			
Pulseless dorsalis pedis artery	Yes	57.5	< 0.0001	46.2	< 0.0001	66.8	< 0.0001	na	na
	No	19.4		30.1		26.3			
Pulseless posterior tibial artery	Yes	56.0	< 0.0001	43.8	< 0.001	66.5	< 0.0001	na	na
	No	21.6		33.3		29.6			
**Modifiable cardiovascular risk factors**									
History of hypertension	Yes	27.2	ns	37.3	ns	39.4	< 0.05	10.4	ns
	No	30.0		37.7		33.6		9.8	
History of hypercholesterolemia	Yes	26.9	< 0.05	38.9	ns	38.1	ns	9.3	< 0.01
	No	30.2		34.7		37.6		14.5	
Smoking	Current	37.4	< 0.0001	49.1	< 0.0001	54.6	< 0.0001	14.3	< 0.01
	Cessation ≤ 1 year	40.5		57.6		50.5		14.6	
	Cessation > 1 year	33.3		37.3		42.0		10.4	
	No	21.2		30.5		29.5		8.8	
Diabetes	Yes	28.6	ns	38.2	ns	44.2	< 0.0001	11.0	ns
	No	27.3		36.9		35.3		9.9	
Number of risk factors	4	45.7	< 0.0001	68.9	< 0.0001	54.2	< 0.0001	18.8	ns
	3	30.8		38.2		47.7		10.0	
	2	23.6		37.7		34.6		10.1	
	1	33.6		33.7		33.5		0.0	
	0	25.4		22.0		26.7		0.0	
**Measurements at the visit**									
SBP (mmHg)	< 140	25.4	< 0.0001	34.8	< 0.01	33.2	< 0.0001	9.5	< 0.01
	[140–160]	26.4		35.6		38.5		9.1	
	≥ 160	37.2		47.1		50.5		15.9	
LDL cholesterol (mmol/l)	< 2.6	26.4	ns	34.0	ns	34.7	< 0.01	8.9	ns
	(2.6–3.3)	25.0		36.4		33.8		8.1	
	(3.3–4)	27.7		35.7		41.8		12.0	
	≥ 4	29.5		42.9		45.6		9.2	

na, not applicable; ns, not significant; SBP, systolic blood pressure; LDL, low-density lipoprotein; PAD, peripheral arterial disease.

### Prevalence of PAD in the predefined clinical subgroups (univariate analyses)

The subgroups significantly differed for the prevalence of PAD (p <0.0001, Figure 1). PAD affected 37.4% (95% CI: 34.7–40.2) of patients in group 1 and 38.0% (95% CI: 36.0–40.0) of patients in group 2, whereas prevalence of PAD was the lowest (10.4%, 95% CI: 9.1–11.7) in patients with two or more cardiovascular risk factors only.

In each subgroup as in the whole cohort (Table 2), prevalence of PAD was increased in univariate analysis in patients with symptoms of PAD, smoking history, increased number of cardiovascular risk factors or excessive brachial SBP at the visit.

**Figure 2:**
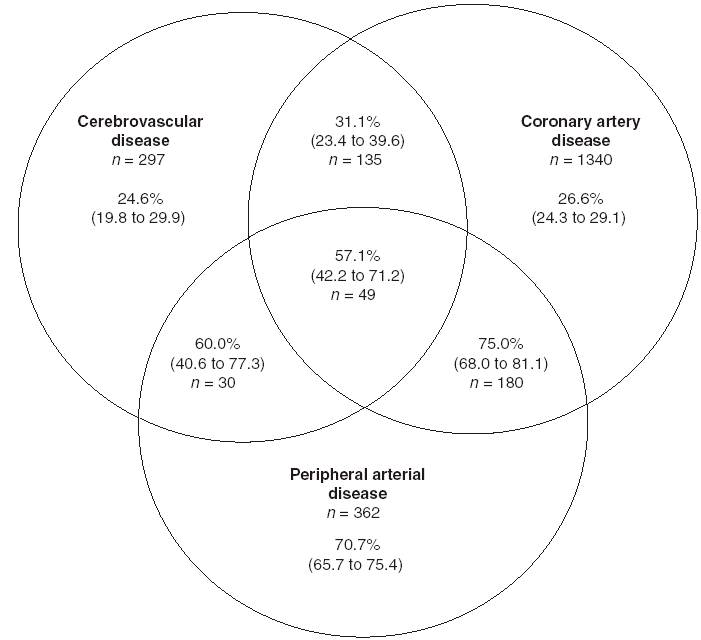
Prevalence (95% confidence interval) of peripheral arterial disease (ankle-brachial index < 0.90) in the subgroup of patients with one or more history of atherothrombotic event according to the localization of the event (*n* = 2393)

In group 2, prevalence of PAD was the highest in patients with past history of symptomatic PAD; the lowest in patients who had experienced cerebrovascular and/or coronary events only (p < 0.0001, Figure 2). In particular, it was 70.7% in those with previous symptomatic PAD and no other vascular localization, whereas it was 26.6% in patients with history of coronary events and no other vascular history and 24.6% in those with history of isolated cerebrovascular events. Prevalence of PAD was increased in patients with history of atherothrombotic events. PAD was also more frequent in patients with history of renovascular hypertension (58.2 vs. 37.4%, p < 0.001).

In group 3, increased prevalence of PAD was observed with smoking habits, excessive brachial SBP at the visit or a history of renovascular hypertension (33.3 vs. 10.3%, p < 0.01). No relationship was found using univariate analyses between an ABI below 0.9 and diabetes, hypertension or the number of cardiovascular risk factors.

### Factors predictive of PAD (multivariate analyses)

The independent variables significantly associated with diagnosis of PAD using the ABI are displayed in Table 3. The statistical model retained the following variables as risk factors for PAD: older age, each symptom suggestive of PAD, cardiovascular risk factors (smoking, diabetes and elevated LDL cholesterol), history of renovascular hypertension and coronary artery disease.

**Table 3:** Independent risk factors for peripheral arterial disease in patients of the cohort excluding those with previously established symptomatic peripheral arterial disease (*n* = 5125). Multivariate logistic regression analysis

Variable vs. reference	Odds ratio (95% CI)	p-value
**Age vs. (55–64) years**		
(64–74) years	1.21 (1.00–1.45)	< 0.05
74 years or older	1.44 (1.18–1.75)	< 0.001
**Sign or symptom suggestive of PAD vs. absent**		
Intermittent claudication	3.73 (3.00–4.63)	< 0.0001
Other leg pain while walking	1.69 (1.42–2.00)	< 0.0001
Pulseless dorsalis pedis artery	2.86 (2.40–3.42)	< 0.0001
Pulseless tibial artery	1.95 (1.61–2.37)	< 0.0001
**Cardiovascular risk factor vs. absent**		
Currently smoking	1.98 (1.62–2.43)	< 0.0001
Formerly smoking (cessation > 1 year)	1.38 (1.15–1.66)	< 0.001
Formerly smoking (cessation ≤ 1 year)	2.48 (1.79–3.42)	< 0.0001
Diabetes	1.24 (1.06–1.44)	< 0.01
LDL ≥ 3.3 mmol/l*	1.81 (1.23–2.66)	< 0.01
**History of atherothrombotic event vs. absent**		
Renovascular hypertension	2.21 (1.17–4.17)	< 0.05
Angina pectoris/coronary artery angioplasty or bypass grafting	1.42 (1.21–1.67)	< 0.0001

* Value at the latest measurement. PAD, peripheral arterial disease; CI, confidence interval.

## Discussion

Peripheral arterial disease in high-risk patients of 55 years or more was highly prevalent (27.8%) and easily detected by a simple ABI measurement in primary care practice. Intermittent claudication arising out of lack of one pulse in the lower limbs, smoking, diabetes and renovascular hypertension (renal artery stenosis) were the main factors predictive of low ABI. On the basis of these results, we recommend to screen for low ABI, in the primary care setting, at least patients presenting with these factors.

Prevalence of PAD ranged from 10.4% in patients with high-risk cardiovascular profile only, to approximately 38% in patients with symptoms suggestive of PAD and more than 25% in patients with previous history of cerebrovascular or coronary disease. Low ABI (below 0.75) was observed in < 3% of patients with multiple risk factors only, but in 17–20% in the other subgroups. It may be noted that PAD was diagnosed in only 46.2–68.8% of patients with a pulseless dorsalis pedis or posterior tibial artery. This underlines the uncertainty of such clinical examination and the necessity of Doppler ultrasound examination.

Prevalence differed depending on localization of previous atherothrombotic events. It was already high (24.6–31.1%) in patients who had experienced cerebrovascular and/or coronary events. It was much higher in patients with past history of symptomatic PAD (57.1–75.0%), but not as high, however, as one would have expected. This was probably because 70.8% of patients with previously established PAD and an ABI of 0.9 or above had undergone a revascularization procedure and some patients were diagnosed in the past based on clinical examination only. Similar results were obtained in the Agatha study (27).

Moreover, not all cardiovascular risk factors influenced the presence of PAD. PAD was more frequent in patients with smoking habits or diabetes and, to a lower extent, in those with history of abdominal aortic aneurysm, renal hypertension or atherothrombotic event. It may be worth noting that cardiovascular risk factors were less predictive of PAD than measurements of arterial blood pressure or LDL cholesterol.

A large number of general practitioners (over 1200) and patients (nearly 6000), well-distributed throughout the territory, participated in the study. Overall, the results are consistent with those of the other large studies in the primary care setting. Prevalence of PAD has been evaluated using the ABI at 18–19% in subjects aged above 55 or 65 years in UK (28), Germany (29) and the Netherlands (20) and at 27–29% in patients with selected vascular risk factors in France (23) and North America (22).

To our knowledge, this was the first prevalence study that took into account all types of high-risk profiles. This allowed deep exploration of the prevalence of PAD and of predictive factors. In particular, although combining history and laboratory values, the cardiovascular risk factors were well characterised, including modifiable and unmodifiable variables. Cardiovascular treatments were, however, not recorded in the study, rendering difficult any analysis of the modifiable risk factors in the population (i.e. hypertension, hypercholesterolemia and diabetes) and the possible impact on ABI measurement. Besides, the well-known poor sensitivity of clinical examination to diagnose PAD may have underestimated the results in patients in two subgroups, those with signs or symptoms suggestive of PAD and those with history of established symptomatic PAD.

In this observational study, diagnosis of PAD was based mainly on the ABI measurement, which is the recommended standard for diagnosis (1). As much as one primary prevention patient out of 10, and one secondary prevention patient out of four, had an ABI below 0.9. Systematic ABI measurement should be performed in order that none of the patients with atypical symptoms and none of those who are asymptomatic, but who are at high risk suffer loss of opportunity. Obviously diagnosis of PAD can be performed by general practitioners. ABI measurement is easily performed, not excessively time-consuming and inexpensive. Further investigations or invasive procedures are reserved for a small minority of patients. Much of the ‘best medical treatment’ can also be implemented in the primary care (30).

However, there is still under diagnosis of PAD and insufficient management of patients with PAD in primary care, in France (31) as in other developed countries (1). Given the very high prevalence of PAD we found in high-risk patients and the cardiovascular morbidity and mortality associated with PAD (2,3), undoubtedly better public and health professional awareness would help preserve individual cardiovascular health and achieve public health goals.
